# Bulk RNA-Seq Analysis Reveals Differentially Expressed Genes Associated with Lateral Branch Angle in Peanut

**DOI:** 10.3390/genes13050841

**Published:** 2022-05-08

**Authors:** Naveed Ahmad, Lei Hou, Junjie Ma, Ximeng Zhou, Han Xia, Mingxiao Wang, Soraya Leal-Bertioli, Shuzhen Zhao, Ruizheng Tian, Jiaowen Pan, Changsheng Li, Aiqin Li, David Bertioli, Xingjun Wang, Chuanzhi Zhao

**Affiliations:** 1 Institute of Crop Germplasm Resources (Institute of Biotechnology), Shandong Academy of Agricultural Sciences, Shandong Provincial Key Laboratory of Crop Genetic Improvement, Ecology and Physiology, Jinan 250100, China; naveedjlau@gmail.com (N.A.); houlei9042@163.com (L.H.); ma_junjie96@163.com (J.M.); coldxia@126.com (H.X.); zhaoshuzhen51@126.com (S.Z.); aarongood@163.com (R.T.); jwpan01@126.com (J.P.); shzyxgs@126.com (C.L.); liaiqin1970@163.com (A.L.); xingjunw@hotmail.com (X.W.); 2 College of Life Sciences, Shandong Normal University, Jinan 250014, China; ximengzhou1995@163.com (X.Z.); wangmx0122@163.com (M.W.); 3 Center for Applied Genetic Technologies, University of Georgia, Athens, GA 30602, USA; sorayabertioli@gmail.com (S.L.-B.); djbertioli@gmail.com (D.B.); 4 Department of Plant Pathology, University of Georgia, Athens, GA 31793, USA; 5 Department of Crop and Soil Science, University of Georgia, Athens, GA 30602, USA

**Keywords:** bulk RNA-sequencing (BR-seq), branch angle, gravitropism, plant hormones, alternative splicing, peanut

## Abstract

Lateral branch angle (LBA), or branch habit, is one of the most important agronomic traits in peanut. To date, the underlying molecular mechanisms of LBA have not been elucidated in peanut. To acquire the differentially expressed genes (DEGs) related to LBA, a TI population was constructed through the hybridization of a bunch-type peanut variety Tifrunner and prostrate-type Ipadur. We report the identification of DEGs related to LBA by sequencing two RNA pools, which were composed of 45 F_3_ lines showing an extreme opposite bunch and prostrate phenotype. We propose to name this approach Bulk RNA-sequencing (BR-seq) as applied to several plant species. Through BR-seq analysis, a total of 3083 differentially expressed genes (DEGs) were identified, including 13 gravitropism-related DEGs, 22 plant hormone-related DEGs, and 55 transcription factors-encoding DEGs. Furthermore, we also identified commonly expressed alternatively spliced (AS) transcripts, of which skipped exon (SE) and retained intron (RI) were most abundant in the prostrate and bunch-type peanut. AS isoforms between prostrate and bunch peanut highlighted important clues to further understand the post-transcriptional regulatory mechanisms of branch angle regulation. Our findings provide not only important insights into the landscape of the regulatory pathway involved in branch angle formation but also present practical information for peanut molecular breeding in the future.

## 1. Introduction

The plant architecture is generally determined by plant height, bud outgrowth, initiation of axillary meristems, and differences in branching growth [1,2]. The branching pattern directly influences peanut physiology, yield, and crop management. In addition, the branching habit of peanut is a major factor in agricultural technology applications, including mechanical culture and control of diseases [3]. The branching habit not only influences the penetration of peanut pegs to produce pods but also affects planting density per unit area. The angle between the main stem and the first lateral branch is a key factor of plant architecture [4,5]. The branch angles were shown to regulate the plant’s ability to absorb light and generate different responses against external factors [6]. Previously, the genetic basis of plant architecture and branch angle control mechanisms have been partially demonstrated in the model plant *Arabidopsis* and rice [2,6]. Similarly, the branching habit in peanut using interspecific QTL mapping was also studied [7]. Enormous efforts have been made to identify genes involving the regulation of branch angle in other plants, such as Lazy1 (LA1), Tiller Angle Control1 (TAC1), and Prostrate Growth1 (PROG1) [8,9,10]. However, the molecular mechanisms determining the branch angles in peanut remain to be elucidated.

Peanut (*Arachis hypogaea* L.) is a commercially important crop worldwide. It is the second-largest cultivated grain legume crop [11]. The cultivated peanut variety is derived from the combination of two diploid progenitors of *Arachis ipaensis* and *Arachis duranensis*. The nutritional value of peanut is not only rich in proteins (20–40%), lipids (40–60%), and carbohydrates (10–20%) [12], but is also rich in vitamins, minerals, and antioxidants [13]. The branching patterns of peanut are generally categorized into four types, including prostrate, spreading, bunch, and erect [14]. For classifying the habit of peanut growth, the index of plant type (IOPT) defined by the ratio of the longest branch of the first two lateral branches to the length of the main axis is also used. The IOPT index is approximately 2.0 for the prostrate type and 1.5 for the spreading type, whereas 1.1–1.2 is for the erect and bunch types [15]. Recently, progress has been made on the identification of QTLs using quantitative locus mapping for understanding plant height regulation in peanut. For example, the assembly of a high-density genetic linkage map was constructed, which resulted in the identification of 18 QTLs for plant height using a recombinant inbred line (RIL) population [16]. Another study revealed 11 QTLs for controlling the main stem height and four QTLs for the lateral branch angle in peanut using an RIL population [5]. Alongside the agronomic significance, the genetic process governing peanut branching is still not evident. Therefore, it is important to study the underlying molecular mechanisms that control the onset of branch angle in peanut.

**Figure 1:**
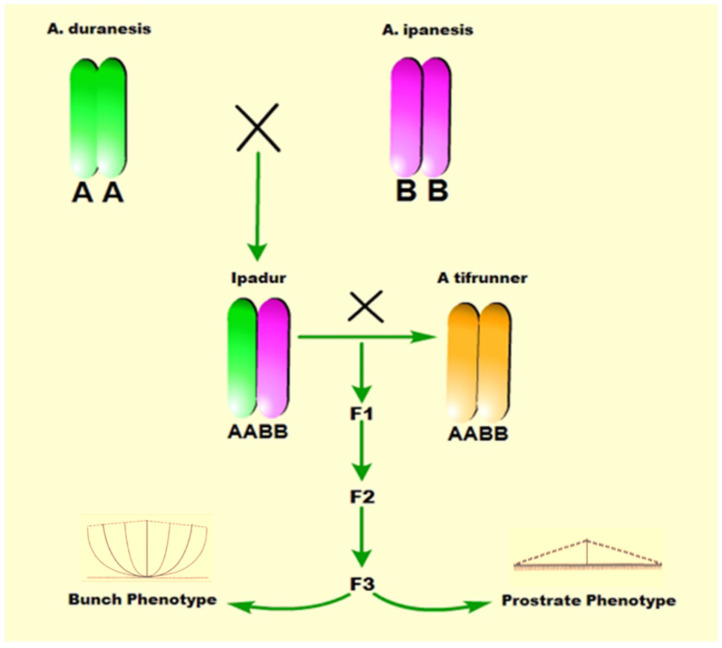
The schematic diagram of generating F3 progenies of TI population obtained from the hybridization of the synthetic tetraploid ‘Ipadur’ (male parent) and tetraploid cultivar *A. hypogea*, variety Tifrunner (female parent). The synthetic tetraploid parent ‘Ipadur’ demonstrated prostrate habit contrasting with tifrunner’ which showed bunch habit, whereas their F3 lines in T1 population developed both prostrate and bunch phenotype.

Over the last two decades, high-throughput transcriptome sequencing has effectively elucidated the genetic control mechanisms involved in plant architecture, including branch angle regulation. In particular, the cDNA microarray profiles of olive plants revealed approximately 2252 differentially expressed genes (DEGs) in different breeding lines [17]. A total of 5908 DEGs were identified from two oilseed rape lines that showed substantial branch angle variations using RNA-seq [18]. A large number of these DEGs were involved in auxin and brassinosteroid signaling pathways [18]. Many highly expressed genes were linked with membrane and cell wall in a comparative transcriptome analysis between columnar and standard growth habits of apple trees [19,20]. The transcriptome analysis of *LA1-*mutant maize and wild-type identified important DEGs related to auxin transport, auxin response, and light signaling pathways [21]. However, the molecular regulatory mechanism of peanut branch angle formation has not been elucidated yet. In the present study, we aimed to decipher the molecular regulatory mechanisms that control branch angle formation using the bulked-RNA-seq approach in prostrate and bunch peanut (Figure 1). The assembly and annotation of the transcriptome data combined with the analysis of the DEGs highlight new insights into the regulatory network of peanut branch angle regulation. In addition, this study also enriches the genomic resources for developing new cultivars with diverse growth habits using marker-assisted selection (MAS) in the future.

## 2. Materials and Methods

### 2.1. Plant Materials, Sampling and Lateral Branch Angle Measurement

The TI population was derived from the hybridization of a bunch-type peanut ‘Tifrunner’ as the female parent with a synthetic prostrate-type peanut ‘Ipadur’ as the male parent. The F_3_ lines from this population were used in this study for transcriptome analysis. For each line, twenty plants were planted in the experimental field at Biotechnology Research Center, Shandong Academy of Agricultural Sciences, Jinan, China. The old seedlings of the bunch and prostrate peanut lines with a growing period of approximately 120 days were used for RNA-seq and expression analysis. The seedling growth time was calculated according to the days after planting (DAP) method. The field conditions were followed according to the standard agricultural practices. The lateral branch angle (LBA) was determined by measuring the angle of the first branch to the main stem, and the graphs were prepared using GraphPad Prism 8.0 software. According to the phenotypic variations, 23 lines showing homozygous prostrate and 22 lines showing homozygous bunch were used for constructing the bulked RNA libraries of prostrate (Ah-Pros) and bunch (Ah-bun) peanut, respectively.

### 2.2. RNA Extraction, Library Preparation and RNA Sequencing

The RNA extraction was performed from the junction of the first lateral meristem and main stem tissues of the prostrate and bunch peanut using Trizol Reagent (TaKaRa, Inc., Dalian, China) following the manufacturer’s protocol. The RNA was examined on 1% agarose gel using 1 μg of RNA from each sample. The integrity and purity of the RNA were assessed with an RNA Nano 6000 Assay Kit of the Bioanalyzer 2100 system (Agilent Technologies, Santa Clara, CA, USA) and NanoPhotometer^®^ spectrophotometer (IMPLEN, Westlake Village, CA, USA). Following the manufacturer’s instructions, sequencing libraries were prepared with the NEBNext^®^ UltraTM RNA Library Prep Kit for Illumina^®^ (NEB, Ipswich, MA, USA). Poly-T oligo-connected magnetic beads were used to isolate the mRNA from the total RNA. The random hexamer oligos and M-MuLV reverse transcriptase (RNase H-) enzyme were used to synthesize the first strand of cDNA. Subsequently, the second strand of cDNA was synthesized with the help of DNA polymerases I and RNase H. The library fragments were purified using the AMPure XP method (Beckman Coulter, Beverly, Brea, CA, USA) in order to choose cDNA fragments with a desired length of 250–300 bp. The quality of the library was screened with the help of the Agilent Bioanalyzer 2100 system. The library was sequenced on the Illumina Novaseq platform, and around 150 bp pair-end reads were generated.

### 2.3. Bulk RNA-Seq Data Analysis

Raw reads were initially interpreted using in-house Perl scripts. Clean reads were retrieved by removing the adapter-containing reads, ploy-N-containing reads, and low-quality reads from the raw reads. The clean reads were measured with the parameters of Q20, Q30, and GC content. The annotation files of the reference genome and gene model were accessed from the genome database directly. The Hisat2 v2.0.5 software package was used to build the index of the reference genome, and then paired-end clean reads were aligned to the reference genome of Arachis hypogaea cv. Tifrunner (https://www.peanutbase.org/data/public/Arachis_hypogaea/ (accessed on 12 September 2021) using Hisat2 v2.0.5.

### 2.4. Novel Transcripts Prediction

The mapped reads of each sample were configured using StringTie (v1.3.3b) [22], following a reference-based approach. The StringTie interface is built on a new network flow algorithm to assemble and quantitate full-length transcripts that display multiple splice variants for each gene locus. The new transcript structure annotation information, such as gene, transcript, and exon, were predicted. After the new transcript assembly, we performed the functional annotation of these new transcripts with databases such as Pfam, SUPERFAMILY, GO, KEGG, etc.

### 2.5. Identification of DEGs

The differentially expressed genes between prostrate and bunch peanut were identified using the DESeq2 R module (1.16.1) [23]. The false discovery rate was regulated by adjusting the *p*-values through Benjamini and Hochberg’s method. An adjusted *p*-value < 0.05 and absolute fold change of 2 were used as the setpoint for differentially expressed genes. The TFs were determined by the PlantTFDB (http://pl://ntfdb.bio.uni-potsdam.de/v3.0/ (accessed on 20 September 2021)) using TF family characteristics.

### 2.6. GO and KEGG Enrichment Analysis

DEGs were subjected to GO enrichment analysis using the clusterProfiler R package. The classification of the GO terms linked with the corrected P-value of less than 0.05 were assumed to be considerably enriched [24]. Furthermore, KEGG pathway analysis was carried out by mapping DEGs to the KEGG database (http://www.genome.jp/kegg// (accessed on 20 September 2021). The enrichment of DEGs in the KEGG pathways was carried out using clusterProfiler R.

### 2.7. Differential Alternative Splicing, SNP and Variant Sites Analysis

Alternative splicing events (AS) consisting of the skipped exon, retained intron, alternative 5′ splice site, alternative 3′splice site, and mutually exclusive exon were investigated in the prostrate and bunch-type samples. The most significant and novel AS events expressed were extracted using rMATS (3.2.5) [25]. In addition, the software package of rMATS also determined the AS event in which the existing exon or intron was included by the inclusion junction counts (IJC), and the exon or intron was skipped by the skipping junction counts (SJC). The quantification of the expressed spliced isoforms was confirmed if the values of IJC and SJC ≥ 1. The evaluation of the differential alternative splicing events was determined by calculating the difference in exons/introns inclusion ratio (|IncLevelDifference|≥ 0.1 and *p* ≤ 0.05) between the prostrate and bunch peanut. SNP calling between the prostrate and bunch-type peanut was carried out using GATK2 (v3.7) software. The GATK standard filter method in combination with additional parameters such as (cluster:3; WindowSize:35; QD < 2.0 o; FS > 30.0; DP < 10 and SnpEff software package was utilized for functional annotation of the differentially expressed variable sites.

### 2.8. Validation of RNA-Seq Data Using qRT-PCR

The expression level of 10 selected DEGs was confirmed using a qRT-PCR assay. For this purpose, the total RNA content was isolated from the junction of the first lateral meristem, and the main stem samples of the prostrate and bunch peanut were used for high-throughput RNA sequencing. The first-strand cDNA templates were synthesized using 1 µg of total RNA with the help of the PrimeScript II reverse transcriptase system (TaKaRa). The gene-specific primers were constructed using Primer3 software. The qRT-PCR experiment was performed on the Real-Time System (ABI7500, Applied Biosystems, Waltham, MA, USA) using SYBR Green PCR Premix HS Taq (TaKaRa). The qRT-PCR reaction was carried out in a total of 20 μL volume with the following thermal cycles: 95 °C for 30 s followed by 40 cycles of 95 °C for 5 s and 60 °C for 30 s. The qRT-PCR reaction for each DEG was performed in three independent biological replicates along with the actin gene as an internal reference gene. The relative expression level of each transcript between bunch and prostrate peanut was calculated according to the 2^−^^ΔΔCt^ method.

## 3. Results

### 3.1. Phenotypic Variations in Lateral Branch Angle of the Bunch and Prostrate Peanut

**Figure 2:**
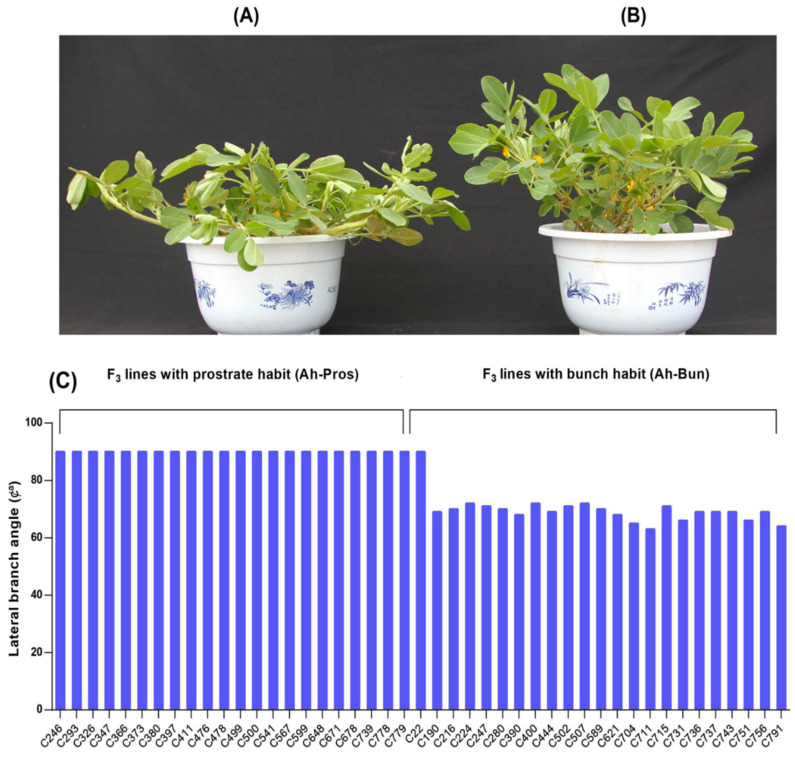
Phenotypic variations of prostrate and bunch growth habits in the F3 lines of TI population (**A**) phenotype of bunch-type peanut demonstrating bunch growth habit (**B**) Phenotype of prostrate-type peanut demonstrating prostrate growth habit (**C**) Variations in lateral branch angle of prostrate and bunch F3 lines selected for construction of RNA pools.

Phenotypic differences were observed in the lateral branch angle of the F_3_ lines of population TI (Tifrunner × Ipadur). Among the 45 selected F_3_ lines for bulked RNA analysis, 22 lines showed the type of bunch growth habit, whereas 23 lines were found with the prostrate phenotype (Figure 2A,B). The lateral branch angle of the 23 lines showed a similar phenotype to their male parents, Ipadur, with a branch angle of 90 degrees, demonstrating the prostrate phenotype (Figure 2C). Similarly, the lateral branch angle of the 22 lines ranged from 63 degrees (C711) to 72 degrees (C224, C400, and C507), demonstrating a curved upward/bunch branching pattern, similar to their parental Tifrunner line (Figure 2C).

### 3.2. Bulked-RNA-Seq and De Novo Assembly

The mRNA from the bunch and prostrate peanut samples (the junction of the first lateral meristem and main stem tissues) was extracted, and a total of six libraries were constructed. Libraries were sequenced using the Illumina Novaseq platform. More than 200 million high-quality reads with an average of ~52 million reads from each sample were aligned to the reference genome. Following the removal of the adaptor sequences, low-quality and N-containing reads of 51.7 and 46.2 million clean reads from the prostrate and bunch-type peanut were obtained. The GC contents of the six tested libraries were in the range of 43.6% to 44.33% (Table 1). Approximately 44.5 and 43.4 million clean reads in each library matched the reference genome with a perfect average mapping ratio of 85.7% and 94.08%, respectively (Supplementary Table S1). Furthermore, the proportions of reads in the exon, intron, and intergenic regions of the genome were also counted. The proportions of reads that aligned to the exons were relatively high, i.e., 77.4% and 75.4% for the prostrate and bunch-type peanut, respectively. The distribution of sequencing reads of all samples in the genomic region is shown in (Supplementary Table S1).

### 3.3. New Transcript Annotation

A total of 5783 new transcripts from the clean reads mapped to the reference genome of the cultivated peanut (*Arachis hypogaea* L.) were annotated as new transcripts by using StringTie software (v1.3.3b). The average length of the new transcripts was 1817 bp, ranging from 200 to 23,092 bp, distributed evenly on different chromosomes (Supplementary Table S2). After the new transcript assembly, we performed the functional annotation of new transcripts with databases such as Pfam, SUPERFAMILY, GO, KEGG, etc. The respective Pfam results, chromosome number, functional description, and SUPERFAMILY information are given in (Supplementary Table S2). GO analysis classified the new transcripts into three functional categories, including molecular function, biological process, and cellular component. It was found that most of the new transcripts were annotated into the biological process category comprising the top two terms of biosynthetic and metabolic process. The most enriched terms in the molecular function category include catalytic activity and binding. In the cellular component category, new transcripts were mostly enriched in the cell part, intracellular part, chloroplast, and mitochondrial membrane part (Supplementary Table S3). Similarly, the KEGG pathways analysis showed that biosynthesis of secondary metabolites, spliceosome, basal transcription factors, metabolic pathways, biosynthesis of amino acids, plant hormone signal transduction, and ribosome biogenesis were enriched (Supplementary Table S4).

### 3.4. Differentially Expressed Genes between Prostrate and Bunch Type Peanut

**Figure 3:**
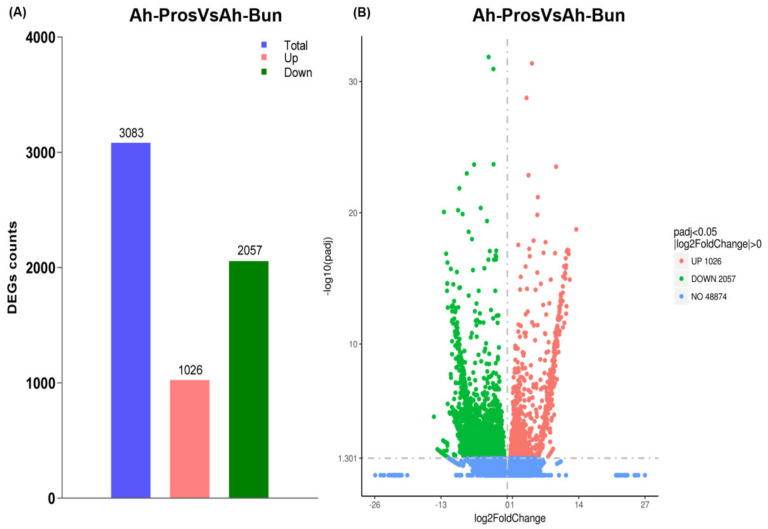
Differentially expressed genes between prostrate and bunch-type peanut (**A**) the statistics of DEG count in prostrate and bunch-type peanut. The blue color represents the total number of DEGs, pink and green color showed up-regulated and down-regulated differential genes, respectively (**B**) The abscissa in the figure represents the fold change of gene expression (log2FoldChange) between prostrate and bunch peanut. The ordinate represents the significance level of the difference in gene expression between two groups (−log10padj or −log10pvalue). The up-regulated genes are represented by red dots, and the down-regulated genes are represented by green dots.

A total of 51,957 genes were detected from all samples, and the gene expression level between the prostrate and bunch-type peanut were further analyzed. In total, 3083 differentially expressed genes (DEGs) were identified, of which,1026 DEGs were up-regulated, and 2057 were down-regulated (Figure 3A; Supplementary Tables S5 and S6). The total numbers of down-regulated DEGs between the prostrate and bunch-type peanut were apparently higher than that of up-regulated genes. The volcano map revealed the visual display of the DEGs between prostrate and bunch-type peanut (Figure 3B). Genes of various signaling and regulatory pathways were preferentially expressed between the prostrate and bunch-type peanut, including NB-ARC, cytochrome P450, auxin response factor, signal peptide peptidase, ABC transporters, Pkinase, PP2C, and leucine-rich repeat (LRRNT), etc. Similarly, the members of different transcription factor families such as MYB DNA-binding TFs, WRKY, AATF leucine zipper-containing domain (AATF-ZIP), MADS-box, and WD-40 TFs were also differentially expressed between prostrate and bunch-type peanut.

### 3.5. Functional Enrichment Analysis of DEGs

**Figure 4:**
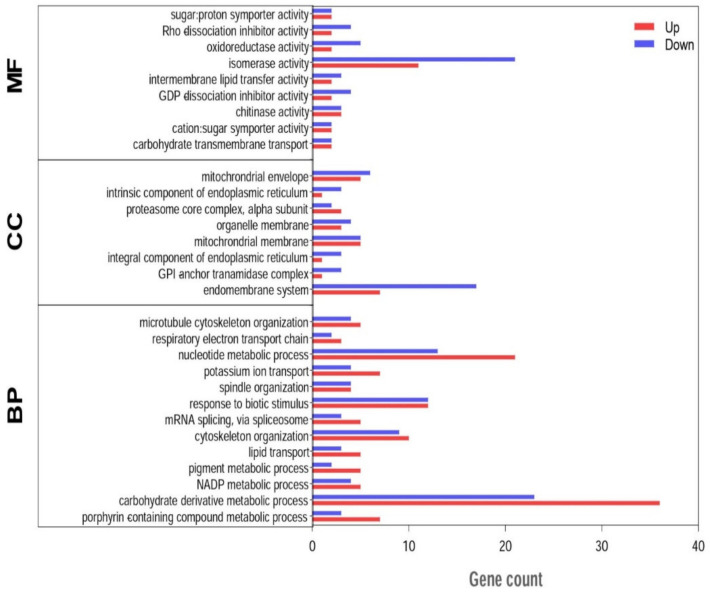
The classification of GO enrichment of DEGs identified in prostrate and bunch-type peanut. The Y-axis represents the number of genes annotated into the GO terms, and the X-axis represents enriched functional categories.

**Figure 5:**
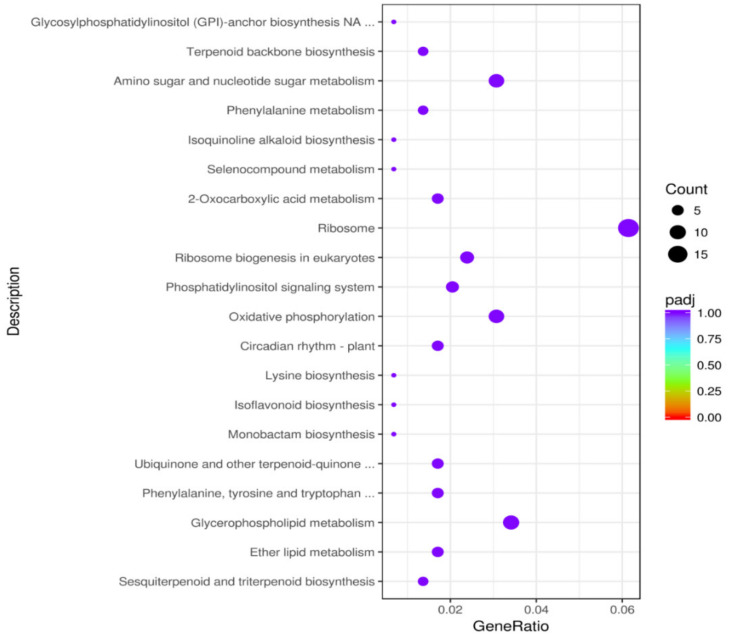
The scatter plot of top 20 KEGG pathways up-regulated in prostrate and bunch peanut. The x-axis represents the ratio of the number of differential genes annotated to the KEGG pathway to the total number of differential genes.

According to the GO classification, a total of 1643 DEGs from the prostrate and bunch-type peanut were divided into three parts, including biological process, molecular function, and cellular component. The most enriched terms in the biological process include biosynthetic, metabolic, and cellular processes. The binding and catalytic activities were the most abundant terms in the molecular function category. For the cellular component category, cell, membrane, membrane part, and organelle were mostly enriched. The scatter plot of the GO enrichment, and top 20 enriched terms in prostrate and bunch-type peanut are demonstrated in (Figure 4, Supplementary Table S7). Similarly, the KEGG enrichment results indicated that a wide range of biological pathways, such as circadian rhythm, plant hormone signal transduction, phenylalanine, tyrosine and tryptophan biosynthesis, photosynthesis, tryptophan metabolism, phenylalanine metabolism, and starch and sucrose metabolism were enriched in the prostrate and bunch-type peanut (Supplementary Table S8). Moreover, the functional pathways related to amino acid biosynthesis, isoflavonoid biosynthesis, ether lipid metabolism, and oxidative phosphorylation were also enriched in the top 20 pathways (Figure 5).

### 3.6. Differential Expression of Gravitropism Related Genes

**Figure 6:**
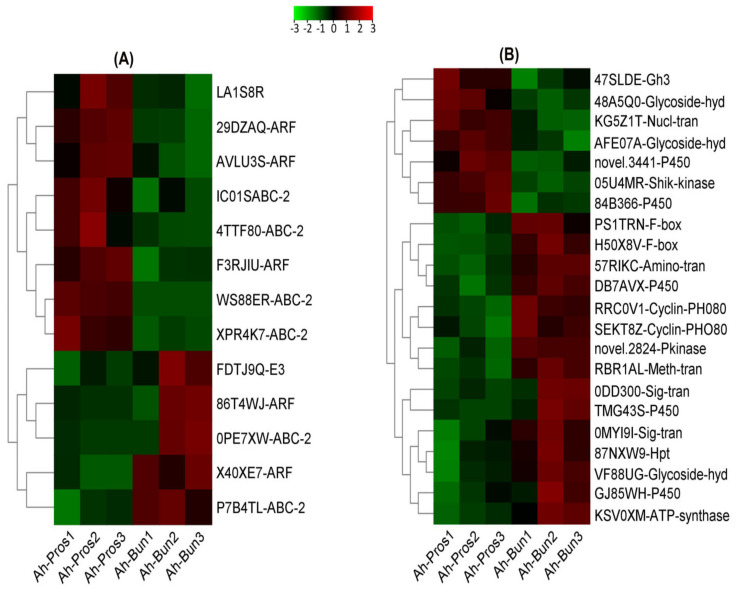
The differential expression pattern of genes involved in the lateral branch angle regulation of prostrate and bunch type peanut (**A**) The expression changes of gravitropism related genes between prostrate and bunch peanut (**B**) The expression changes of genes related to plant hormone and signaling pathways between prostrate and bunch peanut. Scale bar at the top is log2foldchange ratio varying from green (down) to red (up).

The RNA-seq results indicated that several genes related to gravity were differentially expressed between the prostrate and bunch-type peanut. For example, three auxin-responsive factor encoding genes were up-regulated in the prostrate peanut, whereas two auxin-responsive factor encoding genes were abundantly expressed in the bunch-type peanut (Figure 6A; Supplementary Table S9). Previous studies confirmed that genes involved in auxin and brassinosteroid-related pathways are the key regulators of branch angles in plants [18]. Similarly, the expression level of one gene encoding E3 ubiquitin-protein ligase-UBR4 was up-regulated in the bunch-type peanut. E3 ligase is a known gravity-responsive enzyme that specifically promotes the sediment of amyloplasts in *Arabidopsis* [26]. Noticeably, six differentially expressed genes encoding ABC transporter 2 protein family members showed distinct expression levels in the prostrate and bunch-type peanut. Of them, four genes were up-regulated in the prostrate-type peanut while two were preferentially expressed in the bunch-type peanut (Figure 6A; Supplementary Table S9). The expression of genes encoding the ABC transporter 2 protein family was significantly regulated in bermudagrass with erect and prostrate growth habits [27]. These findings suggested that the differential expression of known gravity-related genes may play crucial roles in lateral branch angle regulation in peanut.

### 3.7. Expression of Genes Related to Plant Hormones and Signaling Pathways

The roles of hormonal synthesis and signaling pathways are crucial for plant growth and development. To date, a wide range of hormonal and signaling genes has set the stage to uncover the underlying molecular mechanisms of plant architecture [28,29,30]. Our results also demonstrated significant changes in several genes involved in hormonal biosynthesis in these two types of peanut. For instance, a gene encoding glycoside hydrolase 3 (GH3), which is involved in various plant hormone pathways such as indoleacetic acid (IAA), jasmonic acid (JA), and salicylic acid (SA) [31], was preferentially expressed in prostrate peanut compared with the bunch-type peanut (Figure 6B; Supplementary Table S11). Similarly, genes encoding cytochrome P450 heme-containing enzymes, involved in several hormone metabolism-related pathways, were differentially expressed in the prostrate and bunch-type peanut. Two genes encoding CYP735A1-like cytokinin hydroxylase and CYP45094C1-like protein were up-regulated in the prostrate relative to bunch-type peanut (Figure 6B; Supplementary Table S10). Reportedly, cytokinin hydroxylase regulates trans-zeatin biosynthesis during peanut pod development [32], whereas CYP45094C1 catalyzes the oxidation of jasmonoyl-L-isoleucine (JA-Ile) in the jasmonate-dependant signaling pathway [33]. Comparably, three putative P450 encoding genes were preferentially expressed in the bunch-type compared with prostrate peanut (Figure 6B; Supplementary Table S11). Two genes encoding ent-kaurenoic acid oxidase 1 (CYP701A26), catalyzing gibberellin biosynthesis, were up-regulated in bunch-type peanut [34]. One gene annotated as cytochrome P450711A1 was up-regulated in bunch-type peanut, which catalyzes the strigolactone signaling pathway. Strigolactones are the type of phytohormones that inhibit tillering and shoot branching through the MAX-dependent pathway [35].

To further elucidate the underlying molecular mechanisms and regulatory networks of branch angle formation, the differential expression of genes related to signal transduction pathways were identified in prostrate and bunch peanut. Several signaling genes were differentially expressed in the prostrate and bunch-type peanut. For example, the expression of two F-box encoding genes showed up-regulation in bunch-type compared with prostrate-type peanut (Figure 6B; Supplementary Table S10). Previous studies revealed that F-box genes are involved in several plant growth and development processes, including embryogenesis, senescence, circadian rhythms, light signals, floral development, and seedling development [36]. The expression changes of the two genes encoding signal transduction response regulator, receiver domain was increased in bunch-type peanut. Similarly, many other signaling genes encoding protein kinase-leucine rich repeat, histidine kinase, phosphotransfer (Hpt) domain, and aminotransferase, classI/classII were abundantly expressed in bunch-type relative to prostrate peanut (Figure 6B; Supplementary Table S10). Moreover, one secondary metabolism-related gene encoding o-methyltransferase was up-regulated in the bunch-type, whereas a shikimate kinase/glucokinase gene was up-regulated in prostrate peanut. In bunch peanut, two genes encoding cyclin-PHO80-like regulatory proteins were up-regulated, which promote cell cycle progression [37]. One photosynthesis-related gene encoding ATP synthase, F1 complex, gamma subunit was detected with significant expression level in bunch-type compared with prostrate peanut. In addition, four genes of starch and sucrose metabolism exhibited changed expression levels in prostrate and bunch peanut. Genes encoding the nucleotidyl transferase domain, glycoside hydrolase family 17, and glycoside hydrolase, family 77 were distinctly expressed in the prostrate peanut. However, one glycoside hydrolase family 31 was up-regulated in the bunch-type peanut (Figure 6B; Supplementary Table S10).

### 3.8. Transcription Factors between Prostrate and Bunch Peanut

We identified several transcription factors involved in gravistimulation effects that were differentially expressed between the prostrate and bunch-type peanut (Supplementary Table S11). The expression of several homeobox-containing genes encoding MYB, HALZ, and DDT-like DNA-binding domains and one heat shock factor (HSF)-type DNA-binding were mostly down-regulated in the prostrate-type relative to the bunch peanut. A previous study identified that HSFA2D and two WUSCHEL-RELATED HOMEOBOX genes encoding WOX6/11 were actively involved in regulating rice tiller angle via the LA1-dependant auxin distribution pathway [38]. Similarly, one transcription factor gene encoding the TGA-like domain showed up-regulation in bunch lines, and two AATF leucine zipper-containing domains (AATF-ZIP) showed different expressions, of which one was up-regulated in bunch lines, and the other one showed up-regulation in prostrate peanut (Supplementary Table S11). Furthermore, the expression level of three genes encoding MADS-box transcription factors was observed to be differentially expressed, two of them were up-regulated in prostrate, and one was up-regulated in bunch-type peanut. A total of nine genes encoding WRKY transcription factor were identified, of which five WRKY encoding genes were significantly up-regulated in bunch lines, and four were slightly up-regulated in prostrate peanut. Importantly, most of the WD-40 encoding genes were up-regulated in the bunch-type peanut, while only three WD-40 encoding genes were up-regulated in the prostrate-type peanut (Supplementary Table S11). Together, the identification and expression pattern of these important transcription factors could help us in understanding the regulatory mechanism of branch angle formation in peanut.

### 3.9. The Regulation of AS, SNPs and Multiple Variant Sites between Prostrate and Bunch Peanut

The alternative splicing regulations, including skipped exon (SE), retained intron (RI), alternative 5′ splice site (A5SS), alternative 3′ splice site (A3SS), and mutual exclusive exons (MXE), were analyzed in the prostrate and bunch peanut with the help of rMATS tools. A total of 12,394 differentially expressed AS events were commonly identified, of which the main AS events were SE, RI, and A3SS. The most significant expressed AS events in the prostrate and bunch peanut were found to be in exon skipping (SE) and retained intron (RI) (Supplementary Table S12). The significant alternative splicing events were identified based on the FDR value ≤ 0.05 for splicing differences and differential expression. Interestingly, eight genes corresponding to the skipped exon, including D0K4TI, BT3C4B, Y4TJ2Z, ZSY7EJ, K8QLTQ, W0TS07, X9QMY1, 4B5N5U, and one gene, NTI4MN, corresponding to the retained intron were found to be overlapped with previously identified DEGs.

**Figure 7:**
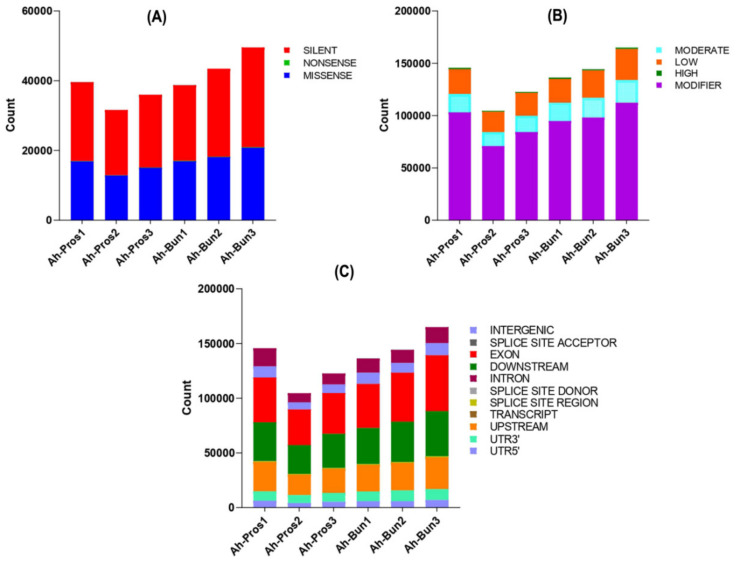
The statistics of SNPs and variant sites identified between prostrate and bunch peanut using GATK tools. (**A**) The identification of variant site function statistics. (**B**) Variant site area statistics, and (**C**) variant site impact statistics. The abscissa in the figure represents the sample name, and the ordinate represents the amount of variations.

Furthermore, GATK tools were used to search for SNPs and variant sites between prostrate and bunch peanut. The variable site function (snp_function) between the two peanut types was statistically graphed from three aspects: silent mutation, missense mutation, and nonsense mutation. The variable site function between prostrate and bunch peanut commonly identified approximately 137,789 silent mutations, 711 nonsense mutations, and 100,491 missense mutations (Figure 7A, Supplementary Table S13). In addition, the impact of mutation sites (snp_impact) confirmed the relative level of phenotypic impact on four levels, high, moderate, low, and modifier. The sum of the impact of mutation sites at the moderate level was 103,290, at the low level it was 144,314, at the high level it was 6774, and at the modifier level it was 563,745 (Figure 7B, Supplementary Table S13). The mutation sites’ regional distribution (snp_region) was also statistically mapped to exon, intron, intergenic, and other gene structure regions. Our results revealed that the highest number of variant sites, i.e., 246,936, was distributed in the exonic region, followed by 203,954, and 146,053 in the downstream region and upstream regions, respectively. Similarly, the intronic, intergenic, UTR-3’ and UTR-5’ regions shared the occurrence of 74,848, 54,741, 52,901, and 34,400 variant sites, respectively. The lowest numbers of variant sites were detected in the transcript region (55), followed by the splice site acceptor region (285), splice site donor region (425), and splice site region (4155) consecutively (Figure 7C, Supplementary Table S13). In summary, the strong evidence of differential AS events in combination with SNP incidence and variant sites laid the basis for understanding the posttranscriptional regulatory mechanism of branch angle formation in peanut.

### 3.10. Validation of DEGs Using qRT-PCR Analysis

**Figure 8:**
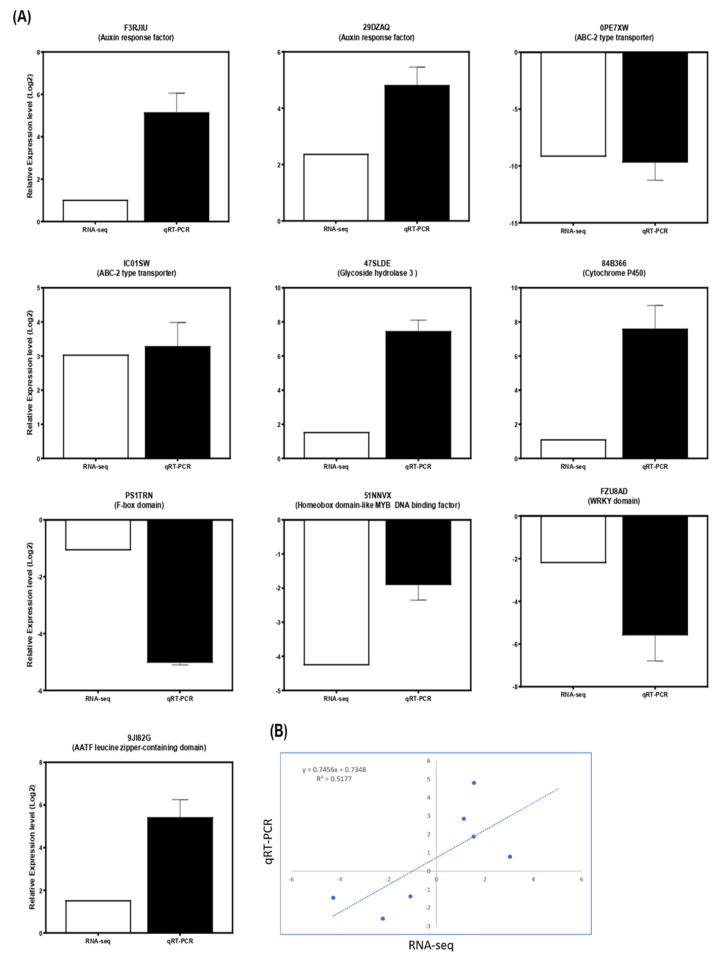
The quantitative real-time PCR analysis of DEGs identified during the onset of branch angle formation in prostrate and bunch-type peanut. (**A**) The expression level of 10 genes related to gravitropism, hormonal and signal transduction and transcription factors encoding genes was validated with qRT-PCR analysis and then compared with RNA-seq data. The data were presented as means of three independent biological replicates, and error bars denote ± SE (*n* = 3). (**B**) The Pearson’s correlation coefficient of a liner regression of the transcripts level ratios between RNA-seq and qRT-PCR data. The correlation of the gene expression was calculated from RNA-seq data (x-axis) and qRT-PCR data (y-axis).

The relative expression level of genes exhibiting diverse expression patterns during the onset of branch angle formation in the prostrate and bunch-type peanut was investigated using Quantitative Real-time PCR (qRT-PCR) analysis to verify the integrity of RNA-seq data. A total of 10 genes were selected, of which four genes related to gravitropic growth, three genes related to plant hormone and signaling pathways, and three transcription factors encoding genes (Figure 8A). Our results indicated that the relative expression levels (log2 prostrate/bunch) of the designated DEGs validated by the qRT-PCR analysis were mostly in line with RNA-seq data. In addition, the reliability of the qRT-PCR data with RNA-seq data was further verified by Pearson’s correlation coefficient of a linear regression analysis in prostrate and bunch peanut, demonstrating 0.517 of the R^2^ squared value (Figure 8B). The primers details are listed in Supplementary Table S14.

## 4. Discussion

Tetraploid cultivated Peanut (2n = 4x = 40) is believed to have originated from the fusion between two diploid species, *Arachis duranensis* (AA) and *Arachis ipaensis* (BB) [39]. In the present study, the mechanism that regulates the angle between the main stem and the first lateral branch in peanut was investigated using the F_3_ lines of population TI (Tifrunner x Ipadur). Bulk RNA-seq was performed using libraries constructed from prostrate and bunch-type peanut. The degree of the vegetative shoot, flower branching, branch angle, and internode elongation are some of the crucial architectural features in peanut [2,40,41]. The phototropism and gravitropism are also considered crucial for the organization of plant architectures [42]. The tropistic growth manner can also maximize the plants’ ability to absorb nutrients/water, photosynthesis, disease resistance, and reproductive cycle [43]. During this study, our transcriptomic data revealed many genes involved in gravitropism that were differentially expressed in prostrate and bunch-type peanut. Among these gravitropism-related genes, several auxin-responsive factors encoding genes were abundantly expressed in prostrate while some were up-regulated in bunch peanut. Previous studies confirmed that genes involved in auxin and brassinosteroid-related pathways are key regulators of branch angles in plants [18,44]. A recent study also showed that several auxin and light signaling-related genes in maize plants were differentially expressed in LA1 mutant and wild-type [21]. Studies also suggested that the regulation of gravitropic response in plants is mainly induced by the asymmetric distribution of auxin resulting in asymmetric organ growth [45,46]. In addition, the suppression of starch metabolism and/or amyloplastic sedimentation in rice mutants revealed decreased gravitropic response and thus resulted in the formation of a larger tiller angle [47,48]. Noticeably, our results further revealed that four ABC transporter 2 encoding genes were up-regulated in prostrate peanut while two of them were up-regulated in bunch peanut. According to another study, the ABC transporter 2 protein-encoding genes were significantly expressed in bermudagrass containing erect and prostrate habits [27]. Our findings proposed that the differential expression of these known gravity-related genes may underlie the regulatory mechanism of lateral branch angle formation and determination of prostrate and bunch growth habits of peanut.

The molecular mechanisms controlling plant architecture involve a complex process of phytohormones and signaling pathways regulation. To date, a wide range of hormonal and signaling genes has set the stage to uncover the underlying molecular mechanisms of plant architecture [49]. Cytokinins are an important class of phytohormones that are involved in cell division regulation and the control of meristem activity [50]. SLs can suppress auxin biosynthesis and inhibit shoot gravitropism in rice [51]. Auxin and BRs may also play roles synergistically during the regulation of plant development [52]. Gibberellin biosynthesis also mediates light and developmental signals by regulating important cellular functions such as cell elongation and division [53]. Our findings also revealed significant changes in several genes involved in plant hormones pathways. For example, a gene encoding GH3, known to be involved in IAA, JA, and SA pathways [31], was up-regulated in prostrate-type peanut. Moreover, two genes encoding CYP735A1-like cytokinin hydroxylase and CYP45094C1-like protein were up-regulated in prostrate peanut. Three putative P450 encoding ent-kaurenoic acid oxidase 1 (CYP701A26) and cytochrome P450711A1 genes were up-regulated in bunch-type peanut. These P450 genes are known to regulate gibberellin biosynthesis and strigolactone signaling pathway in plants [34,35]. Our findings suggested that differential expression of these genes may lead to synergistic effects and could contribute to the underlying mechanism of peanut branch angle regulation.

Several transcription factors, including HD-ZIP (110), HEAT STRESS TFs, genes encoding plant-specific GRAS proteins, HOMEOBOX-containing transcription factors 6, R2R3-type MYB TF family, the helix-loop helix group of proteins, and NAMs encode putative transcription factors containing the NAC domain were involved in the molecular control of shoot gravitropism and auxiliary meristem initiation [38,54,55,56,57,58,59]. Moc1 mutant develops a main peak with no or minimal side branches and a small number of rachis branches and spikes in rice [55]. The moc1 gene is a rice ortholog of the tomato LS and Arabidopsis LAS genes, which promotes the transcriptional regulators of the GRAS family in plants [9,60]. Our findings also indicated that DEGs encode several important transcription factors in prostrate and bunch-type peanut. The expression of several homeobox-containing genes encoding MYB, HALZ, and DDT-like DNA-binding domains and one heat shock factor type were mostly down-regulated in prostrate peanut. Furthermore, one gene encoding TGA-like domain and AATF leucine zipper-containing domains showed up-regulation in bunch-type peanut. The expression level of two MADS-box encoding genes was up-regulated, and one gene was down-regulated in prostrate peanut. Similarly, several WRKY encoding genes were significantly up-regulated in bunch-type peanut, and some were significantly expressed in prostrate peanut. Notably, several WD-40 encoding genes were up-regulated in bunch-type peanut except three WD-40 encoding genes, which were up-regulated in prostrate-type peanut. These results provide both unique and common elements that could play pivotal roles in the signal transduction and functional pathways of gravitropism-related growth leading to the onset of branch angle formation in peanut.

**Figure 9:**
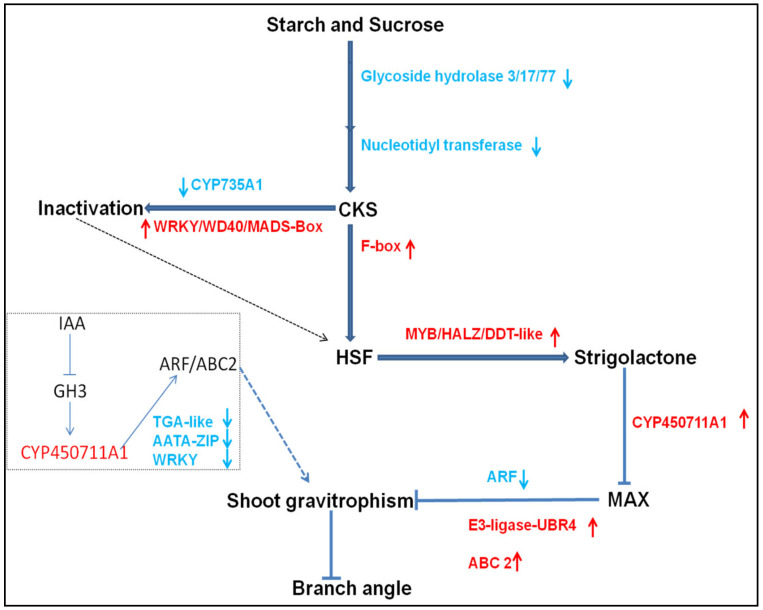
The proposed regulatory network underlying branch angle formation in peanut. Blue arrows demonstrate positive regulation. Red arrows indicate repression. Dotted blue lines demonstrate indirect positive regulation. The inverted blue arrows indicate the inhibition of gravitropic growth. Gravistimulation was affected by starch and sucrose metabolism and regulation of CKs (cytokinins), SLs (Strigolactones) and auxin signaling pathways which may act synergistically in the downstream regulatory pathway of shoot gravitropism in peanut.

Modern transcriptome-wide, genetic, and molecular investigations have shown that posttranscriptional regulation through AS regulates the complicated developmental stages from embryogenesis to the development of a mature plant. [61,62,63]. A wide range of alternatively spliced isoforms have been linked to tissue-specific expression [64], and differential AS events were shown to be involved in plant development [65] and complex environmental responses [66]. In this study, various AS spliced isoforms and AS switches were identified in prostrate and bunch-type peanut. Surprisingly, few genes were demonstrated to undergo skipped exons and retained intron events and were correspondingly overlapping with the previously identified DEGs. It has long been debated that the sophistication of AS regulatory oversight in the fine-tuning of gene expression played a significant role in plant development, their adaptation, and evolution [67,68]. These findings partially corroborated that the regulation of the novel alternative splicing events and their associated factors in prostrate and bunch-type peanut may lead to the reprogramming of gene expression to control variations in plant development. Furthermore, we also could not exclude other post-transcriptional mechanisms that interact with AS, such as microRNAs and post-translational modification, including phosphorylation. Based on these findings, we elucidate a potential regulatory network underlying branch angle formation in peanut. In general, gravistimulation was affected by starch and sucrose metabolism. Several growth hormones and signaling pathways, such as CKs, SLs, and auxin may act synergistically in the regulatory pathway of gravitropism-related growth in peanut (Figure 9). However, a deep understanding is still required to further verify this hypothesis following rational molecular designs and gene editing technologies.

## 5. Conclusions

In this study, we presented a comprehensive transcriptome profiling underlying the regulation of branch angle formation in prostrate and bunch-type peanut. We identified a number of DEGs related to gravitropism and different hormonal mediated signaling pathways, suggesting their important roles in the regulation of branch angle formation in peanut. Our results also revealed that post-transcriptional regulation might also play a vital role in peanut architectural organization.

## Figures and Tables

**Table 1:** Overview of the total read numbers from the F3 lines of C population derived from (Tifrunner × Ipadur) parents.

Sample	Raw Reads	Clean Reads	Clean Bases	Error Rate	Q20	Q30	GC pct
Ah-Pros_1	53,392,552	52,785,702	7.92G	0.03	96.66	90.86	44.04
Ah-Pros_2	41,786,412	41,298,164	6.19G	0.03	97.4	92.48	43.6
Ah-Pros_3	61,942,558	61,235,928	9.19G	0.03	97.36	92.35	44.06
Ah-Bun_1	57,264,296	56,463,890	8.47G	0.03	97.32	92.31	44.23
Ah-Bun_2	41,645,684	41,073,404	6.16G	0.03	97.3	92.27	44.18
Ah-Bun_3	41,798,888	41,291,556	6.19G	0.03	97.35	92.34	44.33

Note: Ah-Pros indicates prostrate habit and Ah-Bun indicates bunch habit of the F3 lines of T1 population. Each sample has three active biological replicates.

## Data Availability

The data associated with this manuscript can be found in the SRA database at NCBI under the BioProject accession number: PRJNA779956 and available online at (https://www.ncbi.nlm.nih.gov/sra/PRJNA779956).

## Supplementary Materials

The following supporting information can be downloaded at: https://www.mdpi.com/article/10.3390/genes13050841/s1. Table S1: The sequencing reads and their distribution in the genomic regions; Table S2: The functional description and SUPERFAMILY information of novel transcript; Table S3: The GO analysis and functional categories of novel transcript; Table S4: The KEGG pathways analysis of novel transcript; Table S5: The list of up-regulated DEGs; Table S6: The list of down-regulated DEGs; Table S7: The statistics of GO term analysis; Table S8: The statistics of KEGG pathway analysis; Table S9: The expression level of gravitropism related genes; Table S10: The expression level of plant hormones and signaling genes; Table S11: The list and expression level of transcription factors-encoding genes; Table S12: The list of differentially expressed AS events; Table S13: The differential characteristics of SNPs and variant sites; Table S14: The list of primers used in qRT-PCR analysis.
